# Thermal Conductivity of Polyamide-6,6/Carbon Nanotube Composites: Effects of Tube Diameter and Polymer Linkage between Tubes

**DOI:** 10.3390/polym11091465

**Published:** 2019-09-07

**Authors:** Mahboube Keshtkar, Nargess Mehdipour, Hossein Eslami

**Affiliations:** Department of Chemistry, College of Sciences, Persian Gulf University, Boushehr 75168, Iran

**Keywords:** polymer nanocomposites, thermal conductivity, Kapitza resistance, reverse nonequilibrium molecular dynamics simulation

## Abstract

Reverse nonequilibrium molecular dynamics simulations were done to quantify the effect of the inclusion of carbon nanotubes (CNTs) in the Polyamide-6,6 matrix on the enhancement in the thermal conductivity of polymer. Two types of systems were simulated; systems in which polymer chains were in contact with a single CNT, and those in which polymer chains were in contact with four CNTs, linked together via polymer linkers at different linkage fractions. In both cases, heat transfer in both perpendicular and parallel (to the CNT axis) directions were studied. To examine the effect of surface curvature (area) on the heat transfer between CNT and polymer, systems containing CNTs of various diameters were simulated. We found a large interfacial thermal resistance at the CNT-polymer boundary. The interfacial thermal resistance depends on the surface area of the CNT (lower resistances were seen at the interface of flatter CNTs) and is reduced by linking CNTs together via polymer chains, with the magnitude of the reduction depending on the linkage fraction. The thermal conductivity of polymer in the perpendicular direction depends on the surface proximity; it is lower at closer distances to the CNT surface and converges to the bulk value at distances as large as 2 nm. The chains at the interface of CNT conduct heat more in the parallel than in the perpendicular directions. The magnitude of this thermal conductivity anisotropy reduces with decreasing the CNT diameter and increasing the linkage fraction. Finally, microscopic parameters obtained from simulations were used to investigate macroscopic thermal conductivities of polymer nanocomposites within the framework of effective medium approximation.

## 1. Introduction

The low thermal conductivity (usually ranging from 0.1 to 0.5 W/(m·K) at room temperature) of polymers limits their use in many engineering applications [1]. For example, in polymer-based electronic systems a higher thermal conductivity of the order of 1 to 30 W/(m·K), is needed to dissipate the waste heat generated during the operation of device [2]. It is known that the addition of highly conductive nanofillers to polymers modifies their thermal/mechanical properties. Due to their excellent resistance to corrosion, light weight, and ease of processing, such polymer nanocomposites are regarded as the new paradigm for materials with diverse applications in electronic, automotive, and aerospace industries, as well as in energy devices [3,4]. In fact, significant alterations in the structural and dynamic properties of polymer occur at very low loadings (≈ 1–5%) of nanofillers, such as graphene nanoplatelets and carbon nanotubes (CNTs). The enormous interfacial area provided by the nanofillers has a large impact on the surrounding polymer matrix, extending to a few radii of gyration of the unperturbed chain.

Among nanofillers, CNTs have attracted considerable attention as ideal fillers due to their high thermal conductivity (≈ 2000–6000 W/(m K) at room temperature). However, even though CNTs have high thermal conductivities, the polymer/CNT nanocomposites have substantially lower thermal conductivities than what would be expected based on a linear law of mixing [5]. In fact, the thermal resistance (Kapitza resistance) at the interface between CNT and polymer is a barrier against heat conduction from CNT to polymer. So far, a significant amount of literature on heat transport in polymer nanocomposites has been devoted to quantifying the thermal resistance at the polymer/filler interface [3,4,5,6,7,8]. In the case of CNT-based nanocomposites, experimental measurements show a moderate increase in the thermal conductivity of polymer matrix (≈ 50–250%) at ≈ 7% of the CNT loading [9,10]. In some cases, even the reports on the thermal resistance at the polymer/filler interface are controversial; the study by Bonnet et al. [11] showed no considerable improvement, and a study by Moisala et al. [12] showed a decrease in the thermal conductivity with the addition of CNTs. 

From a theoretical point of view, the effective medium approximation (EAM) [13] is used to estimate the macroscopic properties of composites through averaging the properties of their constituents. This theory is valid at low filler concentrations, and the estimates of thermal conductivity using this method are much higher than experimental measurements. Further progress in the theoretical estimation of heat transport in the composites was made through the development of acoustic mismatch [14] and diffuse mismatch [15] models. However, both models fail to accurately describe the phonon interfacial scattering process.

Complementary to experimental investigations and theoretical modeling, computer simulation methods have also been conducted to understand the microscopic picture of thermal resistance at the interface. Early molecular dynamics (MD) simulation studies on the heat transfer at solid-polymer junctions include nonequilibrium molecular dynamics (NEMD) simulation of CNT (5, 5)–octane (as a model of polymer) [5], and that of silicon–amorphous polyethylene [16] by Keblinski et al. Further simulation studies in this field consist of heat transfer at the junctions of solids with polymers of more detailed chemical structure at the interface of CNT or graphene sheets [17,18,19,20,21,22,23]. The results of these simulations have revealed a substantial thermal resistance at the polymer/solid interface, depending on the packing of the polymer and chain stretching at the interface as well as the functionalization of CNT/graphene by polymer chains. Although significant information on the mechanism of heat transfer have been obtained in these simulations, there are still unresolved questions on the influence of the molecular nature of polymer/filler interactions on the heat resistance at the CNT/polymer interface.

In this study, we investigated the structure and dynamics of Polyamide-6,6 (PA-6,6) at the interface of CNT (17, 0), CNT (10, 0), and CNT (6, 0) [24]. The aim of this work is to investigate the heat transfer at the interface of PA-6,6/CNTs. The effects of surface curvature, chain orientation, and the linkage of CNTs by polymer chains on the heat transfer will be discussed.

## 2. Theory

**Figure 1:**
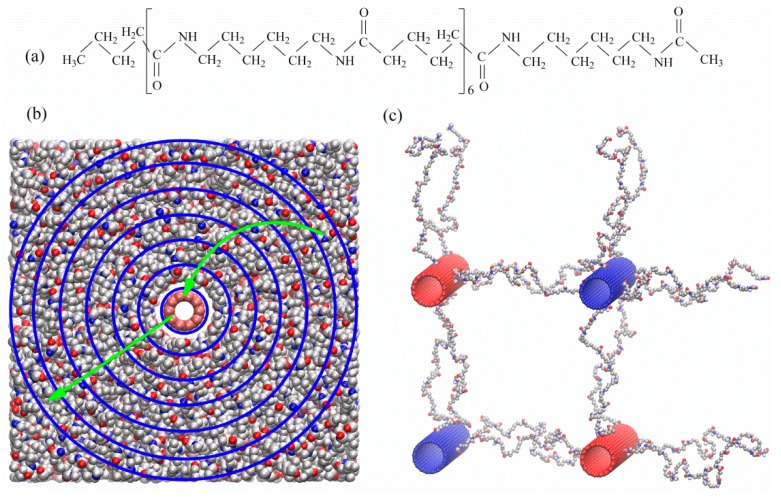
(**a**) Structure of PA-6,6 oligomers simulated in this work. (**b**) A snapshot of a simulation box containing a single CNT (10, 0). The blue, red, grey, and white spheres in the polymer matrix represent N, O, C, and H atoms, respectively. The C atoms of CNT, in the center of box, are shown in red. The curved and straight arrows show the direction of the unphysical heat transfer and the radial heat flow (physical) in the opposite direction, respectively. (**c**) A snapshot of a simulation box containing 4 CNTs (17, 0) laterally linked together via PA-6,6 linkers. For the sake of clarity only CNTs and the linkers are shown. Heat transfer (unphysical) is done from the cold (blue) to the hot red CNTs. In all cases CNTs orient along the z direction.

The reverse nonequilibrium molecular dynamics (RNEMD) technique [25,26,27] was used to calculate the thermal conductivity of PA-6,6 at the CNT interface. According to this method the heat flux is imposed and the resulting force is measured. The heat transfer can be studied in the perpendicular or parallel to the CNT axis directions. The perpendicular heat flow can be studied by dividing the simulation box into a number of cylindrical shells around the CNT. The heat flow is artificially maintained between the CNT and the outermost cylindrical shell. Because the energy is conserved, it flows back through the system (in the radial direction) via a physical transport mechanism. As a result, a temperature gradient develops in the system. A projection of the simulation box on the plane normal to the CNT axis, *xy* plane, which depicts the artificial heat transfer and physical heat flow, is shown in Figure 1b. According to Fourier’s law of heat conduction, the heat flux is connected to the force through the following expression:

(1) $$J\left(r\right)=-\lambda_{⊥}\frac{dT}{dr}$$

where *J*(*r*) is the heat flux in the radial direction, *dT*/*dr* is the temperature gradient along the radial direction (force), and *λ*_⊥_ is the coefficient of thermal conductivity in the perpendicular direction. In the RNEMD method the non-physical heat transfer is performed by exchanging the velocities of atoms in the CNT and the outermost cylindrical shell in Figure 1b. For this purpose, the Cartesian coordinates of velocities of the coldest atom in the hottest cylindrical shell (the C atom in CNT with the smallest velocity) is exchanged with those of the hottest atom in the coldest cylindrical shell (the C atom in the outermost polymer shell with the largest velocity). In the steady state, the average energy flux is expressed as:

(2) $$J\left(r\right)=\frac{1}{2\pi rlt}\underset{\mathrm{transfers}}{\sum }\frac{m}{2}\left(v_{\mathrm{hot}}^{2}-v_{\mathrm{cold}}^{2}\right)=\frac{\Delta \epsilon }{2\pi rlt}$$

where *l* is the length of the CNT, *r* is the radius of the cylindrical shell around the CNT, *m* is the atomic mass, *t* is the duration of the simulation, and subscripts hot and cold refer to the hot and the cold slabs, respectively. In Equation (2), Δ*ϵ*/*t* is the rate of energy transfer between the hot and cold shells and the factor 2*πrl* is the surface area of the cylindrical shell, co-centered with CNT, locating at distance *r* from the CNT axis. From Equations (1) and (2), the thermal conductivity in the perpendicular direction is expressed as:

(3) $$\lambda_{⊥}=-\frac{\Delta \epsilon }{2\pi lt}\frac{1}{\left(dT/dln\left(r\right)\right)}$$

To perform RNEMD simulations of heat transport in the parallel (to the CNT axis) direction the velocity exchange is done in slabs perpendicular to the tube axis. In this case, in Equations (1) and (2), J(r) is replaced with J(z), assuming that the CNT axis orients along the z direction, and the factor 2*πrl* in the denominator of Equation (2) is replaced with $l_{x}l_{y}$ ($l_{x}$ and $l_{y}$ being the dimensions of simulation box along the *x* and *y* directions, respectively). 

## 3. Simulations

We did two sets of simulations for calculating the thermal conductivity of polymer-CNT nanocomposites. In the first set, the PA-6,6 chains consisting of 6 chemical repeat units (see the structure in Figure 1a), were in contact with a single CNT of length 5.983 nm, coinciding with the dimension of the MD simulation box in the *z* direction. Here, the heat transfer in the perpendicular (to the tube axis) direction was studied by exchanging velocities of C atoms of the CNT and those of the outermost cylindrical polymer shell. A snapshot of the simulation box describing the velocity exchange in the perpendicular direction is shown in Figure 1b. 

In the second set, the PA-6,6 chains were in contact with 4 CNTs, linked together by PA-6,6 linkers of fixed length. We have tabulated the details of simulated systems in Table 1. To have the same volume fraction of CNT in the simulation box (as in the case where PA-6,6 chains were in contact with a single CNT), we doubled the size of the simulation box in the x and y directions. The structure of the linkers is close to that of the free PA-6,6 chain; only the terminal methyl and butyl groups of PA-6,6 chains in Figure 1a are removed and the terminal carbonyl C atoms are grafted to CNTs The linkers extending in each direction were grafted to nearly equidistant C atoms along the *z* direction and the number of linkers connecting CNTs along *x* and *y* directions were the same. The axes of CNTs linked together via linkers were ≈ 7.3 nm apart (see Table 1) For systems with linked CNTs, the thermal conductivity in the perpendicular direction was studied by exchanging velocities between CNTs A snapshot of the simulation box, showing the hot and cold CNTs, linked together with PA-6,6 linkers, as shown in Figure 1c. In both sets of simulations the thermal conductivities in the parallel to the tube direction were also studied.

To study the effect of surface curvature on the rate of heat transfer at the interface, in both sets, three systems containing CNT (6, 0), CNT (10, 0), and CNT (17, 0), of diameters 0.475 nm, 0.786 nm, and 1.333 nm, respectively, were simulated. The force field for CNT was the empirical Brenner-type [28,29] and that for PA-6,6 was a flexible united-atom force field [30], which was shown to predict the thermal conductivity of polymer in close agreement with experiment. Equilibrium MD simulations were done at 350 K and 101.3 kPa over a time window of 20 ns to generate relaxed structures. Starting from the relaxed structures, RNEMD simulations were done at different (velocity) exchange periods. The simulation box was periodic in all dimensions. All simulations were done using the simulation package YASP [31]. The temperature and pressure were kept constant using a Berendsen thermostat and barostat [32]. The time constants for coupling the system to the thermostat and barostat were 0.2 ps and 5.0 ps, respectively. The nonbonded interactions were truncated at 0.90 nm and the neighbors were included if they were closer than 1.0 nm. The Coulombic interactions were calculated using the reaction field method with an effective dielectric constant of 5.5 [33]. The time step was 1.0 fs and during the simulation the trajectories of all atoms were recorded every 1 ps. 

## 4. Results and Discussion

### 4.1. Polymer Structure at the Interface

**Figure 2:**
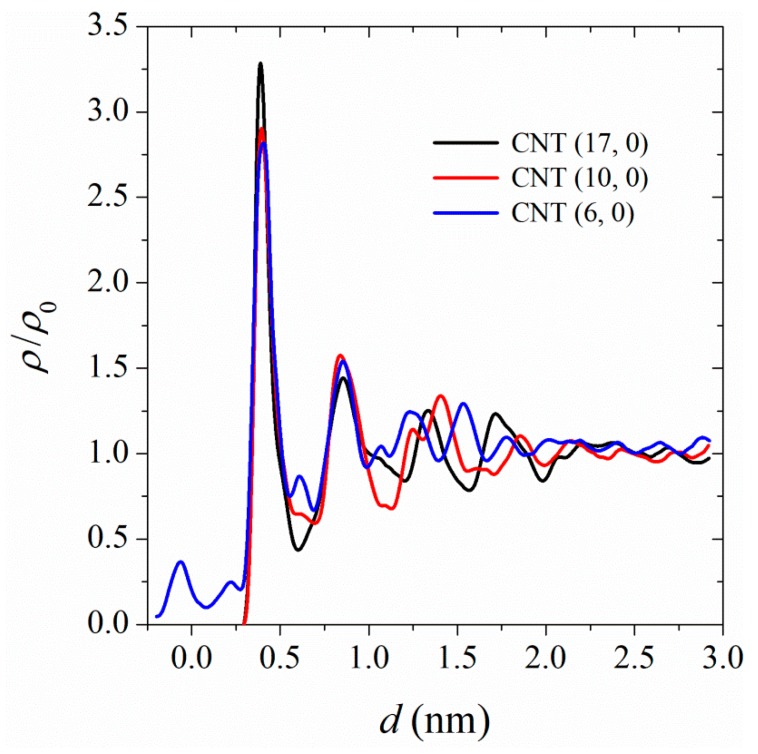
Density profiles for PA-6,6 at the interface of CNT (17, 0), CNT (17, 0), and CNT (17, 0) at 350 K, 101.3 kPa. ρ_0_ is the density of the bulk sample (1100 kg/m^3^).

The microscopic structure of polymer at the CNT interface can be depicted in terms of the density profiles in cylindrical shells around the tube. In Figure 2 we show the density profiles, normalized with the bulk number density, $\rho_{0}$ = 1100 kg/m^3^, for PA-6,6 monomers as a function of their centers-of-mass radial distances from the CNT surface. The polymer at the interface forms ordered layers, which extend to ≈ 2 nm from the CNT surface. The magnitude of the CNT surface effect on the polymer layering at the interface depends on the surface curvature (area). Better structured layers were formed at the interface of the larger-diameter CNT, i.e., CNT (17, 0). The monomers in close vicinity to the CNT (6, 0), whose diameters are very short, wrap around the tube and their center of mass fell inside the tube. In the following sections, we show that heat transfer at the interface depends on the formation of ordered polymer layers.

### 4.2. Temperature Profiles

**Figure 3:**
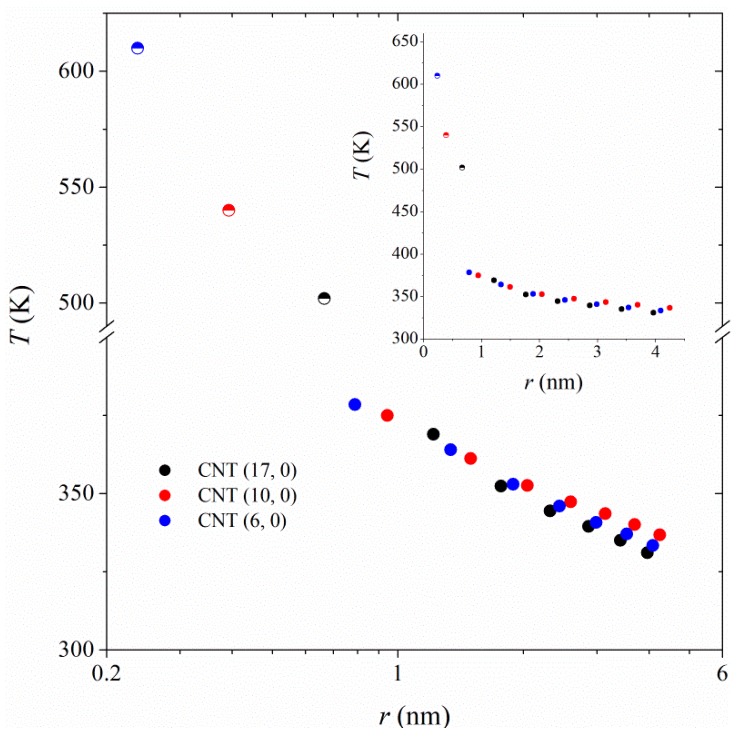
Radial temperature profiles for heat transfer between CNTs and PA-6,6 at 350 K and 101.3 kPa. The type of CNT is shown in the figure’s legend. The horizontal axis represents the distance from the CNT axis. The half-filled symbols represent temperatures and radii of CNTs.

To examine the radial heat flow in the polymer, the simulation box was divided into a number of cylindrical shells (parallel to the CNT axis) of specified thickness. The velocity exchange was done between C atoms of the CNT (the first cylindrical shell) and those of the polymer in the outermost cylindrical shells (see Figure 1b). The shell thickness is adopted as the distance over which the first polymer density profile peak spans, i.e., 0.55 nm (see Figure 2). Averaging the temperature in each cylindrical shell, we plotted the temperature profiles as dT/dln(r) according to Equation (3) in Figure 3. A large temperature jump between the CNT and the polymer film in its vicinity can be seen, which is an indication of a large thermal resistance at the CNT/polymer boundary. The temperature jump is stronger in the case of smaller-diameter CNTs, suggesting weaker heat flow between smaller-diameter CNTs and polymer. 

### 4.3. Perpendicular Thermal Conductivities

**Figure 4:**
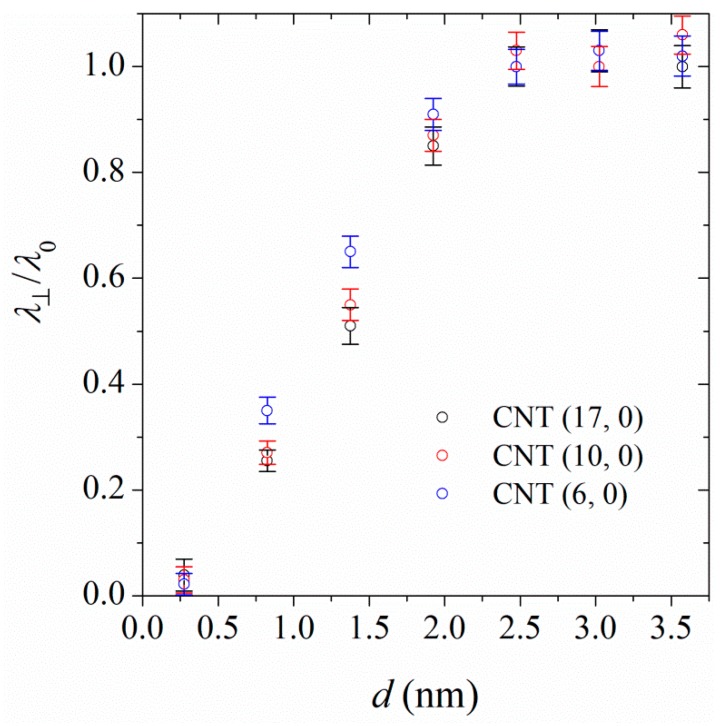
Dependence of perpendicular thermal conductivity, λ_⊥_, of PA-6,6 at the interface of CNT (17, 0), CNT (10, 0), and CNT (6, 0) at 350 K and 101.3 kPa. Here *λ*_0_ is the thermal conductivity of the pure polymer, 0.27 W/(m·K). The values of *λ*_⊥_ at *d* = 0.275 nm are the interfacial thermal conductivities.

To calculate the interfacial thermal conductivities, the energy was assumed to be transferred between the CNT and the first polymer layer, the position of which corresponds to the position of maximum in the first density profile peak (0.55 nm). Therefore, in Equation (2), the surface area corresponds to the surface area of a cylinder with radius *r*_CNT_ + 0.55/2, co-centered with the CNT. The thickness of cylindrical shells is accordingly fixed at 0.55 nm. For heat transfer between polymer shells, the surface area corresponds to the surface area of a cylinder (co-centered with the CNT) with a radius corresponding to the average radii of the layers. We have shown the thermal conductivity of PA-6,6 chains at the interface of CNT (17, 0), CNT (10, 0), and CNT (6, 0) as a function of distance from the CNT surface in Figure 4. Note that the values of *λ*_⊥_ at *d* = 0.275 nm correspond to the interfacial thermal conductivities. The results in Figure 4 show that the perpendicular thermal conductivities at distances longer than 2 nm converge to the corresponding bulk values (*λ*_0_ = 0.27 W/(m·K)). At shorter distances to the CNT, the thermal conductivity decreases with decreasing the distance to the CNT surface. This is due to the formation of organized polymer layers at the CNT interface (see Figure 2). In such organized polymer layers, heat conduction in the perpendicular direction mostly takes place via intermolecular collisions. 

The thermal conductivity falls off suddenly right at the polymer/CNT boundary. The thermal (Kapitza) resistance can be calculated from the temperature jump at the interface and the known flux, according to the following relation:

(4) $$R_{k}=\frac{\Delta T}{J}$$

Our calculated interfacial thermal resistances at the interfaces of PA-6,6 with CNT (17, 0), CNT (10, 0), and CNT (6, 0), correspond to 3 × 10^-8^ m^2^ K/W, 3.9 × 10^-8^ m^2^ K/W, and 4.7 × 10^-8^ m^2^ K/W, respectively. This shows that the interfacial thermal resistance depends on the surface area (diameter) of the CNT; larger resistances are seen at the interface of smaller-diameter CNTs. This result agrees with the findings of Bui et al. [34] on the higher Kapitza resistance at the interface of CNTs (with a diameter of 0.8 nm) compared to graphene sheets of the same volume fraction. As a graphene sheet can be regarded as an infinite-diameter limit of CNT, it is reasonable to accept that the Kapitza resistance of a CNT inversely depends on its diameter (surface area).

### 4.4. Parallel Thermal Conductivities

**Figure 5:**
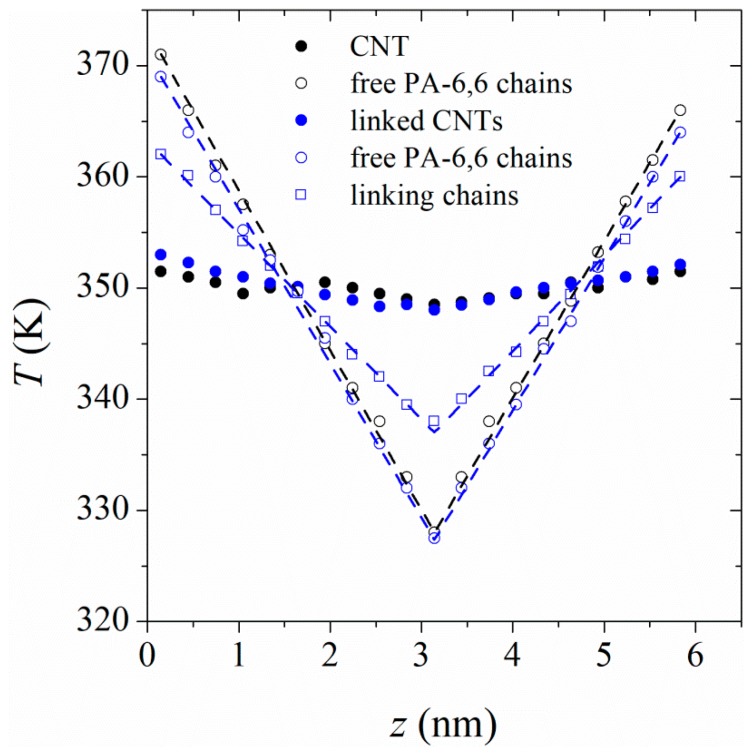
Temperature profiles for heat transfer in the parallel, to the CNT (17, 0) axis, direction. The filled and open markers represent the temperature profile in the CNT and in polymer, respectively. The black symbols belong to the case in which the free PA-6,6 chains are in contact with a single CNT. The blue markers represent a system in which free PA-6,6 are in contact with 4 CNTs linked together with PA-6,6 linkers (linkage fraction = 0.042).

To calculate the thermal conductivities in the parallel to the CNT axis direction, we divided the simulation box along the CNT axis, z direction, into 20 slabs and performed a velocity exchange between the first and the 11th slab. The energy transfer can be either restricted to the exchange of velocities between identical atoms involved in polymer chains, between the two slabs or to include the C atoms of the CNT in the exchange process as well. Previous reports [21,23] in the literature show that both exchange methods lead to nearly identical results for the parallel thermal conductivity of the polymer. Here, we did an unrestricted velocity exchange between the two slabs. The temperature profiles for polymer and CNT for heat transfer in the parallel direction are shown in Figure 5. The results show that while a clear temperature difference between the hottest and coldest regions of the box is observed for polymer, the temperature along the CNT is nearly constant. This is the result of thermal insulation of the CNT from the polymer, as already discussed in terms of the large interfacial thermal resistance. Because of the fact that CNT has a much higher thermal conductivity than the polymer, the temperature gradient in the CNT is very small. This fact also clearly indicates why the two different afore-cited methods of velocity exchange (inclusion/exclusion of CNT in/from the exchange process) give the same results for the parallel thermal conductivity of the polymer.

Our calculated parallel thermal conductivity for PA-6,6 at the interface of CNT (6, 0), CNT (10, 0), and CNT (17, 0) was 0.3 W/(m·K), 0.31 W/(m·K), and 0.33 W/(m·K), respectively. A comparison of the parallel thermal conductivities with the corresponding bulk value, 0.27 W/(m·K) shows that heat transfer in the parallel direction was facilitated at the interface. This can be explained in terms of chain orientation at the interface. Unlike the bulk PA-6,6 sample, in which the chains adopt random orientations, the chains at the interface of CNT are preferentially aligned along the tube axis [24]. We quantified the magnitude of chain ordering in the interphase by plotting the second Legendre polynomial for monomers’ end-to-end vectors. The second Legendre polynomial is defined as:

(5) $$P_{2}\left(d\right)=\frac{1}{2}\left(3\langle u_{1}\cdot u_{2}\rangle {}^{2}-1\right)$$

where *P*_2_(*d*) is the second Legendre polynomial, u_1_ is a unit vector parallel to the CNT axis, **u**_2_ is the monomer’s end-to-end unit vector, and *d* is the monomer’s center-of-mass distance from the CNT surface. Parallel, random, and perpendicular orientations of monomers’ end-to-end vectors to CNT axis correspond to *P*_2_(*d*) = 1, 0, and -0.5, respectively.

**Figure 6:**
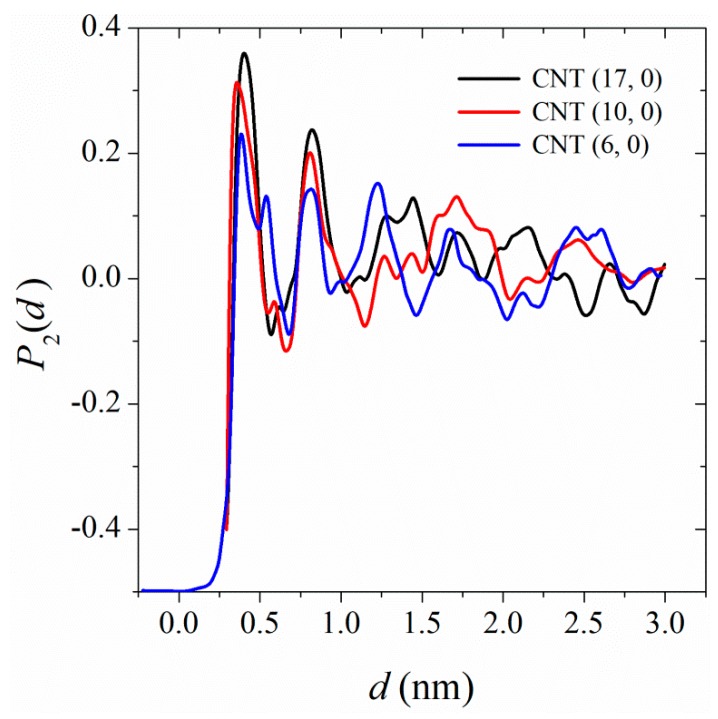
The second Legendre polynomial for the orientation of monomer’s end-to-end vector along the CNT axis as a function of monomer’s center-of-mass distance from the CNT surface.

Figure 6 shows that there is strong perturbation in the chain conformations in the interphase; the chain segments adopt parallel orientations to the CNT axis. The effect is more pronounced for polymer in contact with CNTs of larger surface areas, which explains why the parallel thermal conductivities increases with increasing the CNT surface area. In the present simulation we were not able to calculate local parallel thermal conductivities, i.e., parallel thermal conductivities as a function of distance from the CNT surface, however, one can conclude that local parallel thermal conductivities are larger at closer distances to the CNT surface. This explains the increase in the parallel thermal conductivities of polymer nanocomposites with an increase in the volume fraction of the CNT. In such extended chains, heat transfer occurs through vibration of atoms along the backbone (parallel to the CNT axis). 

### 4.5. Effect of CNT Linkage

**Figure 7:**
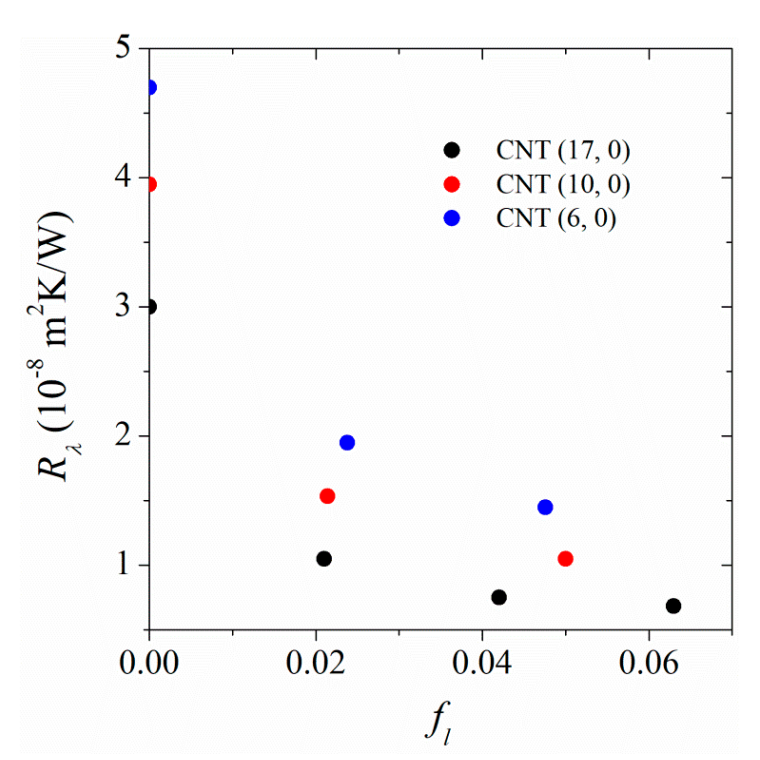
Dependence of CNT-PA-6,6 interfacial thermal resistance (Kapitza resistance) on the linkage fraction.

We performed RNEMD simulations to calculate the thermal conductivities in the parallel and perpendicular directions for systems in which the CNTs are linked together via linkers. The linker has the same structure as the PA-6,6 chains shown in Figure 1a. Only the terminal methyl and butyl groups of PA-6,6 chains were removed and each terminal carbonyl C atom was grafted to the surface of a CNT. During the course of RNEMD simulations, velocity was exchanged between the linked CNTs (see Figure 1c). Temperature profiles were measured in co-centered cylindrical shells around CNTs. The radius of the outer boundary of the outermost cylindrical shell, co-centered with a CNT, extends to 1/16($l_{x}^{2}+l_{y}^{2}$). The shells at identical distances from the axes of both hot CNTs are equivalent; the same is true for shells around cold CNTs. Because of the symmetry, the temperatures in equivalent cylindrical shells were averaged. Finally, two temperature profile curves were plotted (one as a function of distance from the axis of hot CNT and another as a function of distance from the center of cold CNT) and the thermal conductivities at identical distances were averaged. Compared to the case in which free PA-6,6 chains were in contact with a single CNT, a smaller temperature jump between CNT and its closest polymer layer was seen. The magnitude of temperature change at the CNT/polymer interface depends on the linkage fraction, defined as the ratio of number of linked C atoms on the surface of CNT to the total number of C atoms in the CNT. Smaller temperature jumps were observed at higher linkage fractions. In other words, the interfacial thermal resistance (Kapitza resistance) depends on the linkage fraction. Figure 7 shows the Kapitza resistance at the interface of CNT (17, 0) as a function of linkage fraction. The interfacial thermal resistance decreases with increasing the linkage fraction. In fact, the linkers carry energy from the hot tube to the polymer matrix and/or from the polymer matrix to the cold tube. The effect is more pronounced at the interface of larger-diameter CNTs

**Figure 8:**
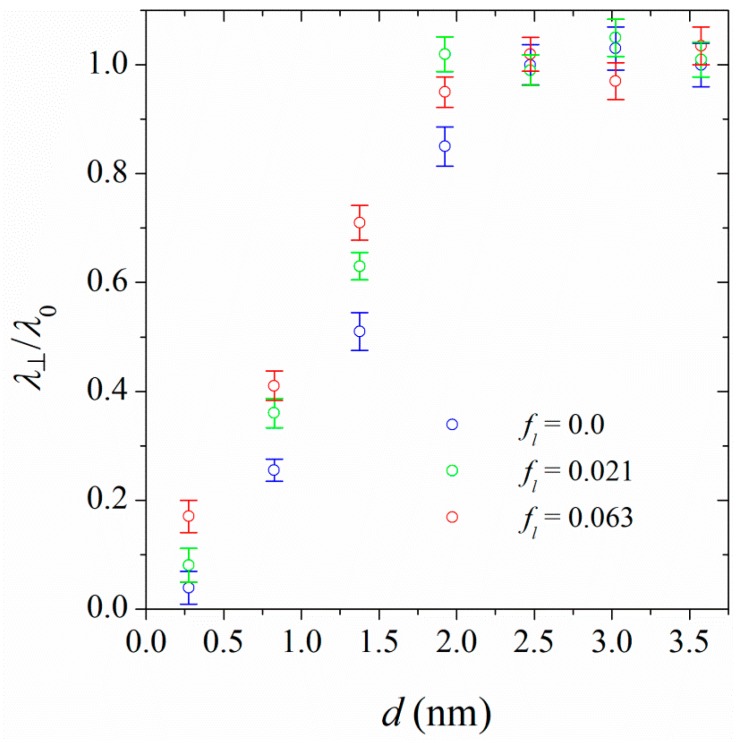
The ratio of perpendicular thermal conductivity, *λ*_⊥_, of PA-6,6 at the interface of CNT (17, 0) to the thermal conductivity of pure polymer, *λ*_0_, at three linkage fractions (shown in the figure’s legend). The points at *d* = 0.275 nm represent interfacial thermal conductivities.

We have also calculated the perpendicular thermal conductivities for PA-6,6 chains at the interface of CNTs. Figure 8 shows the perpendicular thermal conductivity of PA-6,6, normalized with the thermal conductivity of the bulk sample at the interface of CNT (17, 0) as a typical example. The thermal conductivity of polymer at the interface of CNT increases with increasing the distance from the CNT surface. At distances ≈ 2 nm, the perpendicular thermal conductivity converges to the corresponding bulk values. Increasing the linkage fraction increases the perpendicular thermal conductivities at the interface. In other words, the stretched linkers, which act as good conductors, facilitate heat conduction in the polymer. More pronounced effects are seen at distances closer to the CNT (17, 0) surface. 

We performed RNEMD simulations in the parallel to the tube axis. Figure 5 shows the temperature profiles for polymer and CNT. Compared to the results for the case where a single CNT exists in the simulation box, a larger temperature change in the CNT is seen. This means that the conductivity of a functionalized CNT is less than that of a pure CNT, which is in agreement with previous reports in the literature [22,35]. In fact, functionalization introduces defects in the CNT, which act as scattering centers for phonon propagation along the CNT [35]. Moreover, the stretched polymer chains linking the tubes together carry part of the heat in the perpendicular direction. Another noticeable point in the temperature profiles in Figure 5 is the lower slope (temperature gradient) for chains acting as linkers compared to the free chains at the interface of linked CNTs. The stretched polymer chains, linking the CNTs together, have a higher thermal conductivity than free chains. Due to strong anisotropy in their conformations, heat transfer in such stretched chains is performed through vibrations along the chain’s backbone. Penetration of linkers from one slab to the neighboring slabs facilitates heat transfer in the parallel direction. We have also examined the magnitude of anisotropy of heat conduction in the parallel and perpendicular directions in systems containing linked CNTs. The thermal conductivity anisotropy reduces with an increase in the linkage fraction. 

### 4.6. Heat Conduction in Polymer Nanocomposites

The calculated CNT-polymer interfacial thermal resistances can be used as a source for calculating the thermal conductivities of polymer nanocomposites. Due to limitations of the MD simulations, we simulated short-length CNTs at low volume fractions. However, it is known that the thermal conductivity of CNTs depends strongly on their length [29]. To be able to calculate the thermal resistance in polymer nanocomposites, we fitted our calculated thermal resistances with Nan’s [13] effective medium theory (EMA). This model calculates the thermal conductivity for random orientations of micron-sized CNTs in the polymer matrix, using an effective medium approximation.

(6) $$\frac{\Lambda }{\lambda_{0}}=\frac{3+f\left(\beta_{⊥}+\beta_{∥}\right)}{2-f\beta_{x}}$$

where Λ is the thermal conductivity of the composite for random orientations of CNTs, *λ*_0_ is the thermal conductivity of pure PA-6,6, *f* is the volume fraction of CNT, and β_x_ and β_z_ are defined as:

(7) $$\beta_{⊥}=\frac{2\left(\lambda_{⊥}-\lambda_{0}\right)}{\lambda_{⊥}+\lambda_{0}}$$

(8) $$\beta_{∥}=\frac{\lambda_{∥}}{\lambda_{\mathrm{PA}}}-1$$

with

(9) $$\lambda_{⊥}=\frac{\lambda_{\mathrm{CNT}}}{1+\frac{2R_{\lambda }}{D}\lambda_{\mathrm{CNT}}}$$

(10) $$\lambda_{∥}=\frac{\lambda_{\mathrm{CNT}}}{1+\frac{2R_{\lambda }}{l}\lambda_{\mathrm{CNT}}}$$

**Figure 9:**
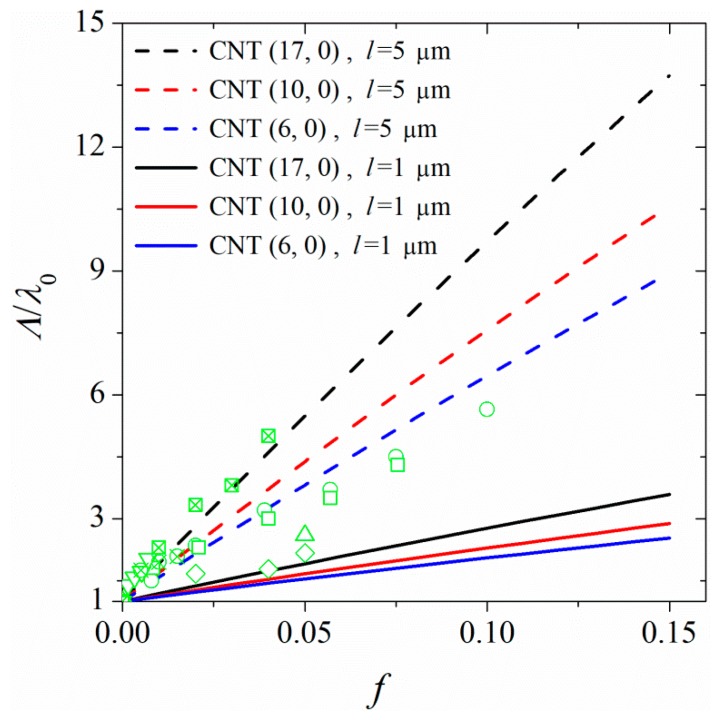
Enhancement in the thermal conductivity of nanocomposite as a function of CNT volume fraction. The full and dashed lines represent the results of EMA modeling for CNTs of length 1 and 5 μm, respectively. The markers represent experimental data by Yu et al. [36], circles; by Yu et al. [9], (squares); by Patti et al. [37], diamonds; by Liu et al. [38], upward triangles; by Deng et al. [39], downward triangles, by Hong and Tai [40] (crossed squares), and by Song and Youn [41] (crossed circles).

In Equations (7–9), *R_λ_* is the interfacial thermal resistance (Kapitza resistance), *λ*_CNT_ is the thermal conductivity of CNT, and D and l are the diameter and length of CNT, respectively. The parameters obtained from our simulations, and used to calculate macroscopic thermal conductivities are reported in Table 2. It is worth mentioning that the volume fractions of CNT in our samples, calculated as the ratio of volume occupied by CNT to the volume of the simulation box, are ≈ 0.004, ≈ 0.01, and ≈ 0.03 for nanocomposites containing CNT (6, 0), CNT (10, 0), and CNT (17, 0), respectively. Here, the volume of CNT is calculated by knowing its diameter and the maximum packing fraction of ordered cylinders (≈ 0.9). As the CNT thermal conductivity is size dependent, we calculated the thermal conductivity of an infinitely long CNT, $\lambda_{\infty ,\mathrm{CNT}}$, by calculating the thermal conductivities of CNTs of different lengths and extrapolating the graph of 1/*λ*_CNT_ vs. 1/ *l* to zero. The results are shown in Table 1. Using the tabulated parameters in Table 1, we calculated the macroscopic thermal conductivity, Λ, for polymer nanocomposites with random orientations of CNTs. Figure 9 shows the thermal conductivities for experimentally relevant (micron) sized CNTs in polymer. We also compared the results of our EAM modeling with experimental data [9,36,37,38,39] in Figure 9. The EAM predictions for micron-sized CNTs are within the range of experimental data [9,36,37,38,39,40,41]. It is worth mentioning that the results presented in Figure 9 are for a CNT/polymer nanocomposite with random orientations of CNTs. 

**Figure 10:**
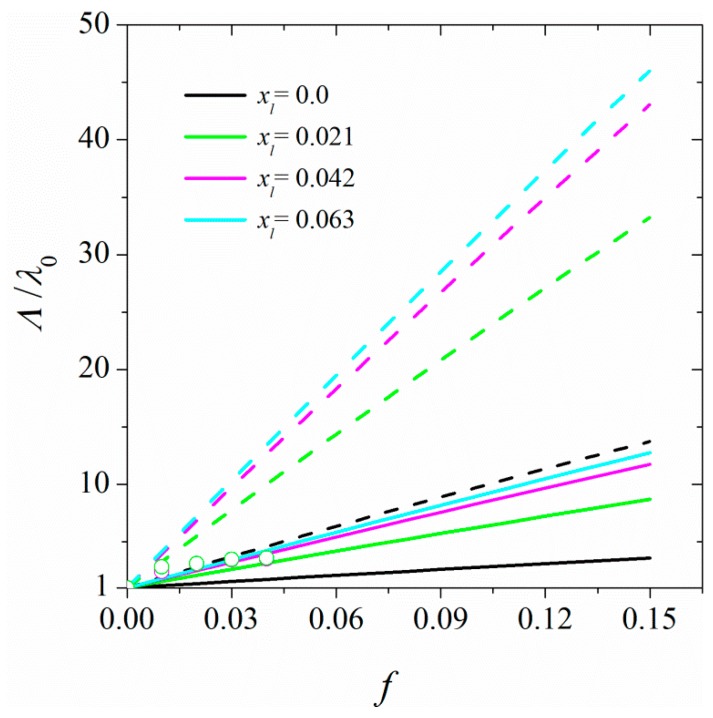
Dependence of the thermal conductivities of nanocomposite, Λ, at different linkage fractions, shown in the figure’s legend, on the volume fraction of CNT (17, 0). The full and dashed curves represent thermal conductivities for CNTs of length 1 and 5 μm, respectively. The markers represent the experimental data by Li et al. [42] on the thermal conductivity of functionalized CNT polymer nanocomposites.

Figure 10 shows the macroscopic thermal conductivities for samples of polymer-linked CNTs at linkage fractions ranging from 0 to ≈ 0.06 and the results are compared with experimental reports of Li et al. [42] on the thermal conductivity of functionalized CNT polymer nanocomposites. A comparison of the results for nanocomposites with linked CNTs and those in which free chains are in contact with randomly oriented CNTs shows that in this case, the linkage between CNTs strongly enhances the thermal conductivity of the nanocomposite with increasing the CNT volume fraction. It is worth mentioning that in this case, we have not taken into account the decrease in the thermal conductivity of the CNT due to defects introduced to CNT by linking chains to its surface.

For a sample of parallel aligned CNTs in polymer, the thermal conductivity of nanocomposite in the parallel, $\Lambda_{∥}$, and perpendicular directions can be expressed as:

(11) $$\Lambda_{∥}=f\lambda_{∥,\mathrm{CNT}}+\left(1-f\right)\lambda_{0}$$

and

(12) $$\Lambda_{⊥}=\lambda_{0}\frac{\lambda_{\mathrm{CNT}}\left(1+\alpha \right)+\lambda_{0}+f\left[\left(1-\alpha \right)-\lambda_{0}\right]}{\lambda_{\mathrm{CNT}}\left(1+\alpha \right)+\lambda_{0}-f\left[\left(1-\alpha \right)-\lambda_{0}\right]}$$

with

(13) $$\alpha =\frac{2R_{\lambda }}{D}$$

**Figure 11:**
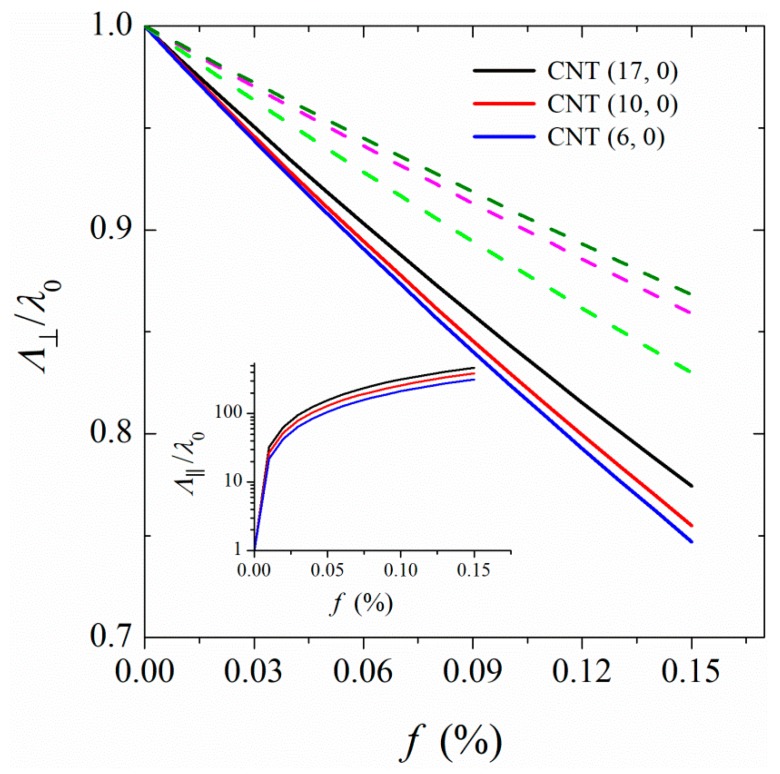
Dependence of the perpendicular thermal conductivities, $\Lambda_{⊥}$ of nanocomposites on the volume fraction of CNTs. The full curves belong to non-linked CNTs and the dashed curves from bottom to up represent the perpendicular thermal conductivities for a nanocomposite containing linked CNTs (17, 0) at linkage fractions 0.021, 0.042, and 0.063. In the inset, the dependence of parallel thermal conductivities, Λ**_∥_** of nanocomposites on the volume fraction of CNTs is shown.

The parallel and perpendicular thermal conductivities of composites as a function of the volume fraction of CNT are shown in Figure 11. A decrease in the perpendicular thermal conductivity with an increase in the volume fraction of CNT was observed. This is understandable because with an increase in the volume fraction of CNT, heat must pass through more CNT/polymer interfaces of high Kapitza resistance. In a nanocomposite containing linked CNTs, the perpendicular thermal conductivity vs. CNT volume fraction curve has a less negative slope than that of a nanocomposite containing non-linked CNTs. This is due to the fact that the linkage of CNTs through polymer chains lowers the Kapitza resistance at the CNT/polymer boundary. Rationally, the perpendicular thermal conductivity of nanocomposite depends on the diameter of the CNT. Because of the larger Kapitza resistance of a smaller-diameter CNT; the thermal conductivity of a nanocomposite filled with a smaller-diameter CNT is lower that filled with larger-diameter CNTs. Contrary to perpendicular thermal conductivity, the thermal conductivity of the composite in the parallel direction grows abruptly with increasing the volume fraction of CNT. As Equation (11) shows, in this case the higher thermal conductivity of CNT compared to that of polymer matrix causes a sudden change in $\Lambda_{∥}$ with a small increase in the amount of CNT in the matrix. Our results on the increase of thermal conductivity in the parallel direction supports the experimental findings of Marconnet et al. [43]. According to their experimental data, at a CNT volume fraction of 0.15, the thermal conductivity in the parallel direction increases by a factor of 15. Our results show a much larger increase in parallel thermal conductivity than that experiment. However, it is worth mentioning that our results refer to samples of perfectly aligned CNTs in polymer, while in an experiment it is not possible to prepare such samples. A combination of both components of thermal conductivity (parallel and perpendicular) leads to an overall increase in the thermal conductivity of the nanocomposite with an increase in the CNT volume fraction (see Figure 9). 

## 5. Conclusions

Although the thermal conductivity of CNTs is very high, their inclusion in polymers does not lead to an enhancement in thermal conductivity, which might be expected based on the linear law of adding the thermal conductivities of both components. The reason is the existence of a large interfacial thermal resistance (Kapitza resistance) at the CNT-polymer boundary. We have shown that the Kapitza resistance depends on the diameter of CNT; it reduces with an increase in the diameter of the CNT. The thermal conductivity of PA-6,6 chains in the perpendicular (to the CNT axis) direction depends on the surface proximity. It is lower at distances closer to the CNT surface and converges to the thermal conductivity of neat polymer at distances as large as 2 nm from the CNT surface. This is due to the formation of organized polymer layers at the CNT interface, in which heat transfer in the perpendicular direction mostly occurs through molecular collisions. Due to the existence of a large Kapitza resistance and lower perpendicular thermal conductivity of polymer at the interface, the thermal conductivity of nanocomposites containing aligned CNTs decreases in the perpendicular direction as the CNT volume fraction increases. Increasing the volume fraction of CNT in such nanocomposites introduces more CNT-boundaries, and hence, larger interfacial thermal resistances that hinder the heat flow in the perpendicular direction. On the other hand, chain orientation at the interface facilities heat transfer in the parallel (along the CNT) direction. The chains at the CNT interface preferentially orient parallel to the CNT axis. Heat transfer in such extended chains mainly occurs through vibrations along the chain backbone, which is faster than the molecular collision mechanism. In other words, in nanocomposite samples containing aligned CNTs, heat transfer in the parallel direction increases with increasing the volume fraction of polymer. As a combination of both factors, the inclusion of CNTs in polymers improves the thermal conductivity of the polymer.

Modifying the interface, through linking the CNTs together with linkers (polymer chains), substantially improves the heat transfer in polymer-CNT composites. The linkers substantially reduce the Kapitza resistance at the CNT-polymer boundary, facilitating heat transfer in the perpendicular direction. The magnitude of decrease in the Kapitza resistance depends on the linkage fraction and the CNT diameter; it reduces more at higher linkage fractions and with increasing the CNT diameter. Both factors, increasing the linkage fraction and increasing the CNT diameter, also facilitate heat transfer in the parallel direction. Therefore, compared to the neat polymer, heat conduction in samples of polymer-linked CNTs improves considerably (depending on the volume fraction of CNT and the linkage fraction). To establish a connection with experimental measurements, we employed microscopic parameters obtained from simulations to investigate macroscopic thermal conductivities of polymer nanocomposites within the framework of effective medium approximation [13]. Our calculations predict experimental measurements on the thermal conductivity of nanocomposites containing randomly oriented micron-sized CNTs in polymers. Inclusion of linkage between CNTs further enhances the thermal conductivity of such nanocomposites. Moreover, it was shown that the thermal conductivity of nanocomposites containing aligned CNTs is reduced in the perpendicular direction, and substantially increases in the parallel direction with increasing the volume fraction of CNT. 

It is worth mentioning that although the simulations/modeling results in this work are done for CNT/PA-6,6 composites, the results are valid for all CNT/polymer nanocomposites. This is because of the fact that compared to CNTs, all polymers have a very low thermal conductivity, and the large polymer-CNT interfacial thermal resistance (because of the large surface to volume ratio) mainly controls the rate of heat transfer in all CNT/polymer nanocomposites.

## Figures and Tables

**Table 1:** Description of systems simulated in this work.^*.^

System	*N_f_*	CNT	*N_l_*	*x_l_*	*l_x_* = *l_y_* (nm)
1	120	CNT (17, 0)	0	0	7.435
2	120	CNT (10, 0)	0	0	7.351
3	120	CNT (6, 0)	0	0	7.306
4	464	CNT (6, 0)	16	0.024	14.623
5	448	CNT (6, 0)	32	0.048	14.646
6	456	CNT (10, 0)	24	0.021	14.700
7	424	CNT (10, 0)	56	0.050	14.753
8	440	CNT (17, 0)	40	0.021	14.868
9	400	CNT (17, 0)	80	0.042	14.885
10	360	CNT (17, 0)	120	0.063	15.944

* In all simulation the CNT length is 5.983 nm, which is equal to the dimension of the simulation box along *z* axis, *l_z_*. *N_f_* is the number of free PA-6,6 chains in the box, *N_l_* is the number of linkers in the simulation box (used to link CNTs together; see Figure 1), and *x_l_* is the linkage fraction defined as the ratio of number of linked C atoms on the surface of CNT to the total number of C atoms in the CNT. The number of C atoms in CNT (6, 0), CNT (10, 0), and CNT (17, 0) are 336, 560, and 952, respectively. The diameters of CNT (6, 0), CNT (10, 0), and CNT (17, 0) are 0.475 nm, 0.786 nm, and 1.333 nm, respectively.

**Table 2:** Parameters used in effective medium approximation (EAM) theory for prediction of thermal conductivities of PA-6,6/CNT nanocomposite.*.

CNT	*R_λ_* (m^2^K/W)	λ_CNT_(W/m·k)	λ_∞,CNT_ (W/m K)	*D* (nm)
CNT(17,0)	3.00×10^-8^	36.9	850	1.333
CNT(10,0)	3.95×10^-8^	30.1	700	0.786
CNT(6,0)	4.71×10^-8^	24.8	570	0.475

* The thermal conductivity of pure PA-6,6, *λ*_0_ = 0.27 W/(m·K). For systems with linked CNTs, the interfacial thermal resistances are given in Figure 8 and the same values of **λ_∞_** for CNTs as reported here were used for parameterization.
